# In-depth assessment of critical access hospital stewardship program adherence to the CDC Core elements in Iowa and Nebraska

**DOI:** 10.1017/ash.2023.273

**Published:** 2023-09-29

**Authors:** Jonathan Ryder, Jeremy Tigh, Andrew Watkins, Jenna Preusker, Daniel Schroeder, Muhammad Salman Ashraf, Trevor Van Schooneveld

## Abstract

**Background:** Critical-access hospitals (CAHs) are required to meet the CDC 7 Core Elements of antimicrobial stewardship programs (ASPs). CAHs have lower adherence to the core elements than larger acute-care hospitals, and literature defining which core-element deficiencies exist within CAHs as well as barriers to adherence is lacking. **Methods:** We evaluated 21 CAH ASPs (5 in Nebraska and 15 in Iowa) that self-identified as potentially deficient in the Core Elements, via self-assessment followed by in-depth interviews with local ASP team members to assess adherence to the CDC Core Elements for ASPs. Core-element compliance was rated as either full (1 point), partial (0.5), or deficient (0), with a maximum score of 7 per ASP. High-priority recommendations to ensure core-element compliance were provided to facilities as written feedback. Self-reported barriers to implementation were thematically categorized. **Results:** Among the 21 CAH ASPs, none fully met all 7 core elements (range, 2.5–6.5), with a median of 5 full core elements met (Fig. 1). Only 6 ASPs (28.6%) had at least partial adherence to each of the 7 core elements. Action (21 of 21, 100%) and leadership commitment (16 of 21, 76.2%) were the core elements with the highest adherence, and accountability (4 of 21, 19%) and education (9 of 21, 42.9%) were the lowest. The most frequent high-priority recommendations were to provide physician and pharmacist leader ASP training (19 of 21, 90.5%), to track antimicrobial stewardship interventions (12 of 21, 57.1%), and to provide or track educational activities (12 of 21, 57.1%) (Fig. 2). One-third of programs were recommended to establish a physician leader. The most commonly self-identified barriers to establishing and maintaining an ASP were a lack of dedicated resources such as time of personnel (15 of 20, 75%), lack of infectious diseases expertise and training (8 of 20, 40%), and electronic medical record limitations (5 of 20, 25%) (Fig. 3). **Conclusions:** CAH ASPs demonstrate several critical gaps in achieving adherence to the CDC Core Elements, primarily in training for physician and pharmacist leaders and providing stewardship-focused education. Further resources and training customized to the issues present in CAH ASPs should be developed.

**Figure:**
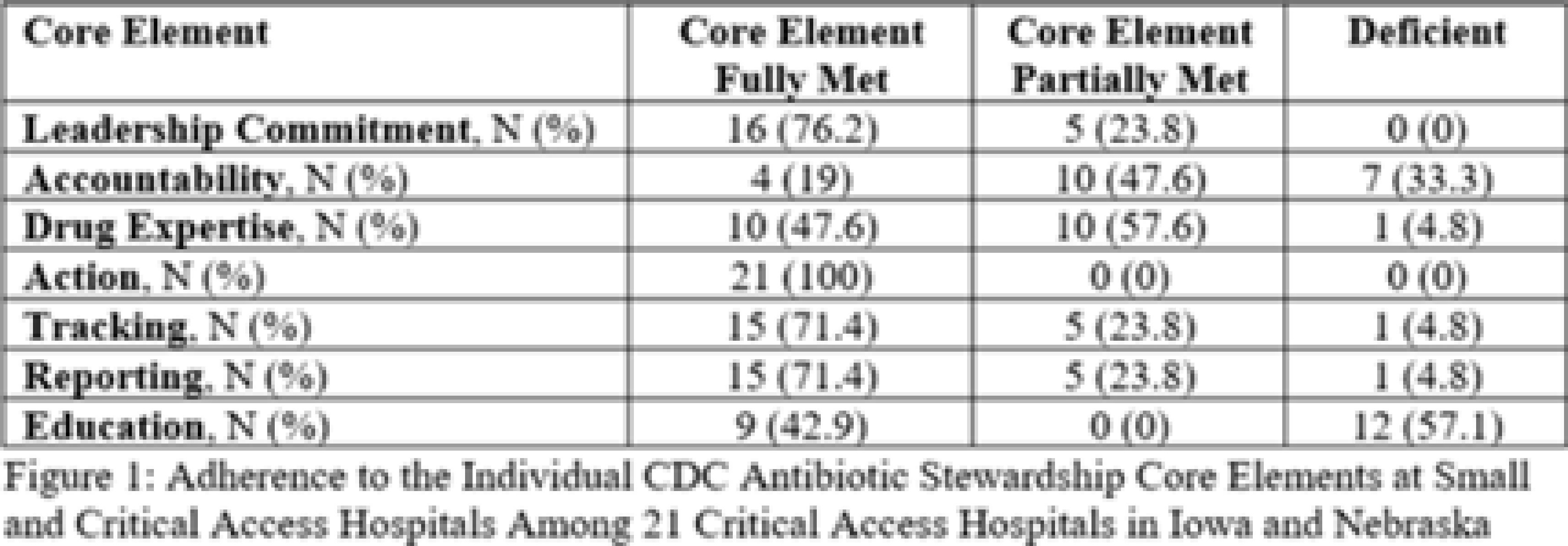

**Figure:**
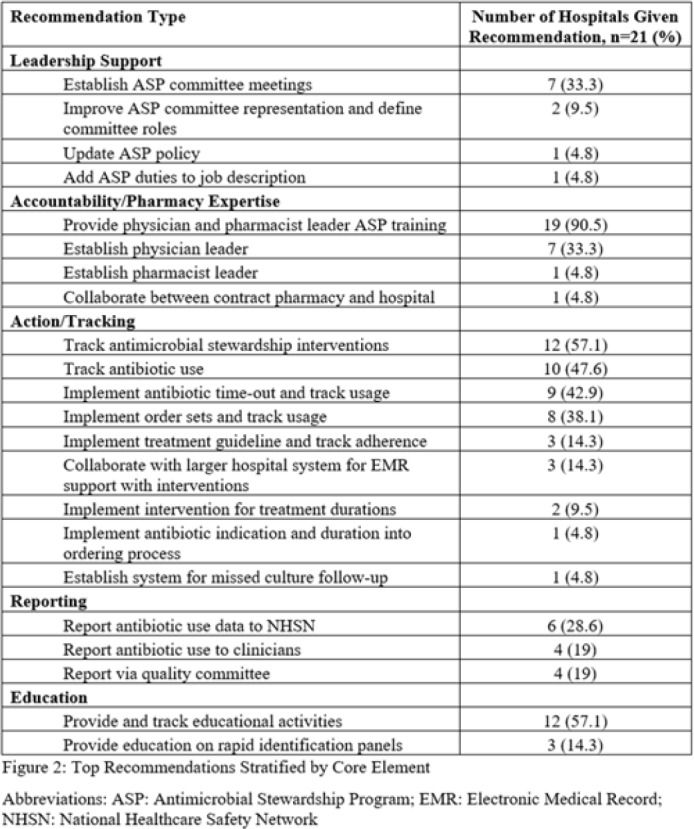

**Figure:**
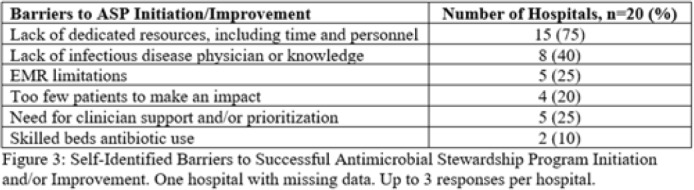

**Disclosures:** None

